# A Pathogenic Missense Variant (c.1617G>A, p.Met539Ile) in *UBA1* Causing Infantile X-Linked Spinal Muscular Atrophy (SMAX2)

**DOI:** 10.3389/fped.2020.00064

**Published:** 2020-02-28

**Authors:** Xin Hua Wang, Lin Mei Zhang, Xue Yang, Shui Zhen Zhou

**Affiliations:** Children's Hospital of Fudan University, Shanghai, China

**Keywords:** infantile X-linked spinal muscular atrophy, SMAX2, gene variant, ubiquitin-like modifier activating enzyme 1, UBA1 gene

## Abstract

**Background:** Infantile X-linked spinal muscular atrophy (SMAX2) is a rare type of spinal muscular atrophy associated with *UBA1* variants.

**Methods:** Clinical imaging and neurophysiological tests were performed on a Chinese patient with SMAX2. Further, focused panel sequencing of *UBA1* was carried out on samples of both the proband and his maternal relatives.

**Results:** The proband, a 4-year-old boy with the SMAX2 phenotype, suffered from reduced exercise capacity since infancy. His other symptoms included speech difficulties, severe nasal tone, reduced distal muscle strength, areflexia, and inadequate sucking ability. The brain MRI of the proband's showed normal results but the electromyography results showed multiple peripheral neurogenic lesions. Five male members of the proband's family were affected with the SMAX2 phenotype. They presented similar symptoms and had experienced a long and autonomous life. Molecular analysis revealed a novel missense variant (c.1617G>A, p.Met539Ile) in the exon 15 of *UBA1*. The proband's mother, as well as grandmother, carried the heterozygous missense *UBA1* variant; whereas, the male patients from the family carried the hemizygotic variant.

**Conclusions:** The affected members in this Chinese family showed unique features such as extended life span, no fractures, and cramps as compared with previously reported SMAX2 cases. The novel missense variant (c.1617G>A (p.Met539Ile) in UBA1 highlights the critical role of this gene in causing SMAX2 phenotype.

## Introduction

Spinal muscular atrophy (SMA) is a group of neuromuscular disorders characterized by the degeneration of the spinal cord motor neurons and muscular atrophy, often leading to early death (1). The most common form of SMA results from motor neuron 1 (*SMN1*) variants in 5q13, which are inherited in an autosomal recessive manner (1, 2). Deficiency or lower levels of the SMN protein expression affects the functional motor neurons in the anterior horn of the spinal cord and causes muscular dystrophy (3).

X-linked SMA is rare and mainly contains two forms (4): X-linked spinal and bulbar muscular atrophy (SMAX1, Kennedy disease, OMIM 313200: Online Mendelian Inheritance in Man 313200) and X-linked infantile spinal muscular atrophy [XL-SMA or SMAX2 (MIM301830)]. SMAX1 is an adult-onset disorder resulting from increase in a polymorphic tandem CAG repeat in the coding regions of androgen receptor (AR) gene on Xq12 (5). SMAX2 is an infantile-onset disease, characterized by hypotonia, areflexia, multiple congenital contractures and infantile deaths, that affect only the males. SMAX2 patients live shorter lives, suffer from respiratory insufficiency due to short and cupped ribs, and present no cognitive damage (4). In addition to these clinical characteristics, degeneration of anterior horn cells and normal SMN1 molecular genetic testing are also typical of SMAX2 cases (6). The genetic analyses of SMAX2 families have identified the ubiquitin-like modifier activating enzyme 1 (UBA1, previously referred to as UBE1) gene located on Xp11.3-q11.2 causing SMAX2 (7). To date, the exon 15 of UBA1 encoding the active adenylation domain (AAD) carries the four SMAX2-causing variants, including three missense (c1617G>T:p.Met539Ile; c1639A>G:p.Ser547Gly; c1670A>T:Glu557Val) and one synonymous variant (c.1731C>T:p.Asn577Asn) (4, 8, 9).

Here, we present five Chinese male SMAX2 patients with partial clinical diagnosis of SMAX2, as ascertained by genetic analysis. However, the clinical manifestations of this case were different from other reported SMAX2 patients. Additionally, gene sequencing of *UBA1* revealed a novel missense variant, c.1617G>A (p.Met539Ile).

## Materials and Methods

### Enrollment of Patients and Clinical Analyses

The proband was a 4-year-old boy, from Jiangsu province, with movement disorder, whose family had approached the Department of Neurology, Children's Hospital, Fudan University, to seek medical attention, in April 2017. The age of symptom-onset, changes in the exercise capacity, and the family history of the proband were investigated, and a comprehensive neurological examination along with brain magnetic resonance imaging (MRI) and electromyography were performed. According to the genetic characteristics of the family history, information regarding the clinical phenotype of other family members with movement disorder, was collected by telephone inquiry. This study was conducted in compliance with all the applicable laws and informed consent requirements.

### Genetic Studies

DNA was isolated from 2 ml of the patient's peripheral blood, using QIAamp DNA Mini Kit. A focused panel sequencing was achieved by Ion Personal Genome Machine (PGMTM) sequencing system and Ion 316TM Chip v2 (10). Using the analysis software provided by the Ion torrent PGMTM server, the sequencing results were analyzed, and the data volume, average sequencing depth, and reference sequence matching degree were obtained. Then, the results were further analyzed by NextGENe software, interpreted in reference to the thousand human gene database (http://www.1000genomes.org) (11, 12) and compared with internal Chinese database which contains about 20,000 cases including samples of children with neuromuscular diseases, such as mental retardation, epilepsy, dyskinesia, autism, etc, and other systemic diseases as well as samples from some parents with normal phenotype in our molecular medical center, the internal database contains both panel sequencing data which includes UBA1 gene and whole exome sequencing data. Following that, the clinical significance of the detected variation was explained using the Single Nucleotide Polymorphism Database (dbSNP) and the Human Gene Mutation Database (HGMD) (13, 14). Also, in order to predict the possible effects of the variants on the protein, the PolyPhen-2, Sorting Intolerant from Tolerant (SIFT) and Mutation-Taster (15, 16) were applied in this study. Additionally, Sanger sequencing of *UBA1* was performed on peripheral blood DNA samples derived from the proband and his maternal relatives, to verify the variant. Mutation surveyor (Soft Genetics, State College, PA) was used to process the sequences against known wild-type control sequences (17).

## Results

### Clinical Phenotypes

**Figure 1:**
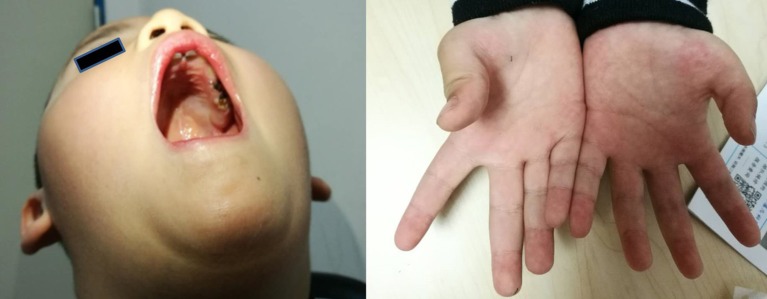
The clinical symptoms of the proband (a 4-year-old boy). Left picture shows that the boy has vaulted arch. Right picture shows that he has flat the nar in both hands.

The main complaint was the slow development of exercise capacity in the proband, since infancy, as compared to his peers. However, this phenomenon was not progressively aggravated. The proband learned to walk independently when he was 18 months old, but the running, climbing, jumping, and coordination abilities were worse than those of his peers. The proband also presented lisp and inadequate sucking ability (for example, in case of eating noodles). Physical examination showed high vaulted arch, difficulty in standing after squatting, normal muscle tension in the limbs, normal proximal muscle strength, distal limb muscle strength slightly weaker than level V, no knee reflex, thumb adduction, and flat the nar (Figure 1). The proband's perceptual development was comparable to that of his peers.

The brain MRI investigations did not reveal any defects of the proband. Electromyography showed multiple peripheral neurogenic lesions (peripheral motor damage with mild demyelination, especially in lower limbs). The amplitude of compound muscle action potential (CMAP) in most of the examined motor nerves, reduced with or without a slight slowing of nerve conduction velocity (NCV). The F wave with the shortest latency from the right ulnar nerve and the bilateral sacral motor nerve were measured. In case of light contractions, the morphology of motor unit potential (MUP) in examined muscle parts was broad and the recruitment of heavy contractions was reduced or slightly reduced.

### Phenotype of Family Members

**Figure 2:**
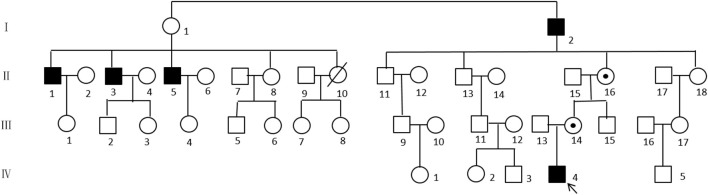
Pedigree of SMAX2 family. Affected males are indicated by black squares and circles are females. Crossed symbols are deceased members. The proband is indicated by the arrow.

We have studied an extended pedigree containing five affected male members, showing SMAX2 phenotypes (Figure 2). These five male patients (individuals I:2, II:1, II:3, II:5, and IV:4) presented symptoms of reduced exercise capacity since infancy, normal cognition, speech difficulties, severe nasal tone and poor sucking ability. The phenomenon was not progressively aggravated, and they had the ability to live on their own as adults. Individual I:2 was still living at the age of 86. The symptoms at the onset of disease in II:1, II:3, and II:5 were similar to those of the proband and the athletic abilities of II:1, II:3, and II:5 were closer to those of a healthy person, mainly showing symptoms of speech difficulties and poor sucking ability.

### Genetic Analysis

A focused panel sequencing of the proband's DNA showed a sequence missense variant in exon 15 of the *UBA1* (NM_003334 in GenBank): c.1617G>A(p.Met539Ile). Sanger sequencing was carried out to verify the missense variant, which was predicted to be benign or tolerable, by Polyphen-2 (score:0.019) or SIFT (score:0.317), but pathogenic, by Mutation Taster (score:1.0). The variant c.1617G>A(p.Met539Ile) has not been included in either the HGMD or the 1000G Project. This candidate is the only record in the internal database of our molecular medical center.

**Figure 3:**
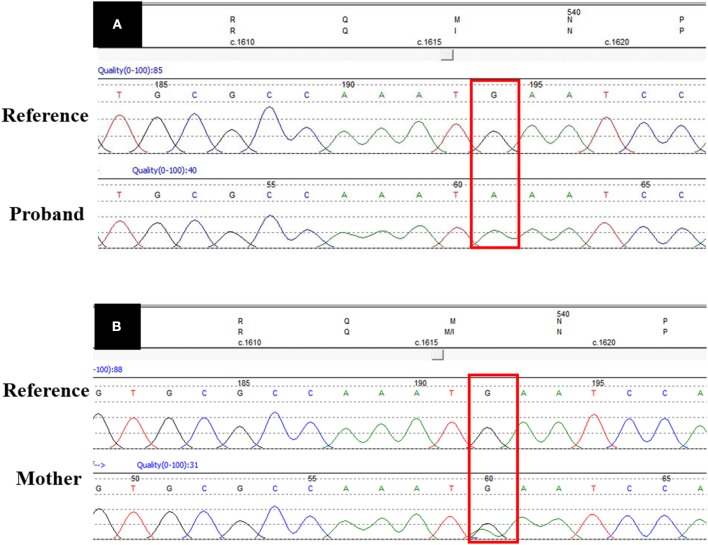
*UBA1* sequence data. **(A)** Shows the pathogenic missense variant (c.1617G>A) in proband's DNA sequence as compared with the reference DNA sequence. **(B)** Shows the heterozygous DNA sequence in the carrier mother. The red box shows the variant site.

Figure 3 shows the missense G-to-A substitution. As shown in Figure 2, the proband's mother (III:14) and grandmother (II1:6) carried the heterozygous missense *UBA1* variant, while, the male patients (I:2 and II:5) carried the hemizygotic variant.

## Discussion

SMAX2 is a severe variant of infantile SMA, inherited in an X-linked manner. The clinical manifestations of SMAX2 are similar to the typical SMA symptoms caused by *SMN1* variants, such as hypotonia, tendon areflexia, and severe myasthenia (1, 18). However, SMAX2 is characterized by facial dysmorphia, cryptorchidism, severe congenital contracture, and fractures. Most patients die of respiratory failure several months after birth (4, 19). Baumbach et al. recommended that the diagnosis of SMAX2 should be based on clinical performances, with congenital hypotonia and areflexia on physical examination, congenital contractures and/or fractures, contractures at birth, and the loss of anterior horn cells in the spinal cord and brain stem, normal *SMN1* molecular sequencing, and male gender in a simple case or an X-linked manner of inheritance in families with multiple patients (18). Females who carry the heterozygous missense have a 50% chance of passing it on to the next generation and males who carry the hemizygotic variant are affected (18).

The incidence of SMAX2 is currently unknown. According to the National Center for Biotechnology Information (NCBI), the clinical and molecular studies of few SMAX2 families are reported so far (4, 9, 19, 20). In Table 1, the clinical features and variant sites are reviewed in detail. All reported cases in the literature suffered a severe muscle weakness after birth and experienced a short lifespan. Among them, one patient reached the age of 13 years and the others died either within a few months or within 2 years after birth. Here, we presented the clinical symptoms and family history of SMAX2 in a Chinese family with a novel variant in the *UBA1*. Genetic analysis revealed a novel hemizygous missense variant (c.1617G>A, p.Met539Ile) in *UBA1* in the proband and other patients as the key factor in the diagnosis of SMAX2. Although our patients showed the typical symptoms of SMAX2 such as areflexia, neurogenic damage, and muscle weakness, their clinical manifestations were significantly different from other cases, with late-onset disease, no progressive aggravation, high-vaulted palate arch, dysarthria, inadequate sucking ability, and a long lifespan.

**Table 1:** Clinical symptoms and gene variant sites of SMAX2 patients reported in literature.

**Patient**	**Described by**	**Hypotonia**	**Areflexia**	**Weakness**	**Contractures**	**Dysmorphic features**	**Inarticulate**	**Weak suck**	**Respiratory distress**	**Intelligence**	**Death**	**EMG**	**Musle biopsy**	**UBA1 variant**
1	Greenberg et al. (20)	Profound	Y	Severe proximal	All four extremities	Myopathic face	NR	NR	NR	NR	18mo	NP	Neurogenic atrophy with some fiber hypertrophy	c.1731C>T p.Asn577Asn
2	Greenberg et al. (20)	Severe	Y	Severe predominantly proximal	Digital	NR	NR	NR	NR	Normal	Alive at age 13 years [Kobayashi et al. (11)]	NP	Neurogenic atrophy with some fiber hypertrophy	c.1731C>T p.Asn577Asn
3	Greenberg et al. (20)	Marked	Y	NR	Fingers, elbows, ankles, knees, hips	NR	NR	NR	NR	NR	5mo	Neurogenic	Neurogenic atrophy	c.1731C>T p.Asn577Asn
4	Greenberg et al. (20)	Severe	Y	NR	Fingers, elbows, knees, hips	Mild potter-like face	NR	NR	Mechanical ventilation	NR	11mo	NP	Neurogenic atrophy with some fiber hypertrophy	c.1731C>T p.Asn577Asn
5	Dlamini et al. (4)	Marked	Y	Global with movement distally in upper limbs, kyphoscoliosis	Fingers, elbows, ankles, knees, hips	Myopathic face	NR	Y	From birth	NR	4mo	Neurogenic	Neurogenic atrophy with some fiber hypertrophy, perivascular inflammation	c. 1670A>Tp.Glu557Val
6	Jedrzejowska (19)	Marked	Y	Severe, lack of the movements of the lower limbs, persisted in proximal muscles of upper limbs	Fingers, wrists, elbows, shoulders, ankles, knees, hips	Myopathic face, tent-shaped open mouth	NR	Y	In the second month of life	Normal	5.5mo	NP	NP	c.1731C>T p.Asn577Asn
7	Ramser et al. (9)	NR	NR	NR	NR	NR	NR	NR	NR	NR	NR	NR	NR	c.1617 G>T p.Met539Ile
8	Ramser et al. (9)	NR	NR	NR	NR	NR	NR	NR	NR	NR	NR	NR	NR	c.1639 A>G p.Ser547Gly
9(I 2)	Our patient	N	Y	Normal proximal, mild weak distal	N	High-vaulted palate arch	mild	Y	N	Normal	Still alive at 86 years now	NP	NP	c.1617G>Ap.Met539Ile
10(II 1)		N	Y	N	N	High-vaulted palate arch	severe	Y	N	Normal	Still alive at 68 years now	NP	NP	NP
11(II 3)		N	Y	N	N	High-vaulted palate arch	severe	Y	N	Normal	Still alive at 65 years now	NP	NP	NP
12(II 5)		N	Y	N	N	High-vaulted palate arch	severe	Y	N	Normal	Still alive at 60 years now	NP	NP	c.1617G>A p.Met539Ile
13(IV4)		N	Y	Normal proximal, mild weak distal	N	High-vaulted palate arch	mild	Y	N	Normal	Alive at 5 years now	Neurogenic	NP	c.1617G>A p.Met539Ile

*N, no; Y, yes; NP, not performed; NR, not reported*.

*UBA1* variants are the only known cause of SMAX2. The *UBA1* encoded protein participates in the ubiquitin conjugation to label the cells for protein degradation using the ubiquitin–proteasome system (UPS) (21). Damage to the UPS has been identified in several neurodegenerative diseases (22). *UBA1* variant may result in an abnormal and complex binding with gigaxonin, which plays an essential role in regulating axonal structure and neuronal maintenance (23). According to Ding et al. the amino-terminal BTB domain of gigaxonin binds to the *UBA1* and the carboxy-terminal kelch repeat domain directly links to the light chain of microtubule-associated protein 1B (MAP1B) (24). Over-expressed gigaxonin results in an increased MAP1B degradation, which further leads to neuron death, while reduction of MAP1B degradation enhances the survival rate of neurons (23). Briefly, *UBA1* missense variant leads to impaired degradation of MAP1B through abnormal gigaxonin, resulting in the death of anterior horn cells in the spinal cord and brain stem.

Only four variants of the *UBA1* have been reported so far, and all of them are localized in exon 15 (4, 9, 19, 20). Balak et al. reported that synonymous C/T substitutions affect the methylation of exon 15 and thereby reduce the *UBA1* expression (7). In this study, we reported the novel base substitution c.1617G>A (p.Met539Ile) in *UBA1* for the first time. Another missense variant c.1617G>T, p.Met539Ile has been reported in another SMAX2 family (9). By comparing these two missense variants, we found that the two different base substitutions occurred at the same site and caused the same amino acid transition (Met is replaced by Ile). As we know, the C-terminus tail of ubiquitin should tightly bind the Adenosine triphosphate (ATP) in a narrow pocket in order to facilitate the acceptance of cysteine in the adjacent second catalytic cysteine half-domain (SCCH) (25). The latest research on the function of variants in *UBA1* suggests that the p.Met539Ile missense variants showed no statistically-reduced adenylation activity, leading to negligible changes in the relative enzymatic activity of UBA1 *in vitro* (7). This may explain why our patients exhibit different clinical performance and have a longer lifespan. The case reported in this study expanded the clinical and neuropathological spectrum related to the variants in the *UBA1* and suggested that an extended diagnosis ought to be considered in affected male infants.

## Conclusion

We have reported a novel missense variant c.1617G>A (p. Met539Ile) in *UBA1* in a Chinese family with SMAX2. Our patients were distinguishable from previously reported cases, with unique features of late-onset disease, no progressive aggravation, vaulted palate arch, dysarthria, reduced sucking ability, and extended lifespan. This study expanded the spectrum of clinical and neuropathological findings associated with variants in *UBA1*.

## Data Availability Statement

The raw data supporting the conclusions of this article will be made available by the authors, without undue reservation, to any qualified researcher.

## Ethics Statement

The studies involving human participants were reviewed and approved by Children's Hospital of Fudan University. Written informed consent to participate in this study was provided by the participants' legal guardian/next of kin. Written informed consent was obtained from the minor(s)' legal guardian/next of kin for the publication of any potentially identifiable images or data included in this article.

## Author Contributions

XW and LZ are the co-first authors. They participated in the study acquisition, analysis and interpretation of data, and drafted the manuscript. XY performed the analysis and interpretation of the clinical data. SZ conceived the idea for the study, designed and supervised the study, interpreted data, drafted and revised the manuscript content. All the authors have read and approved the final manuscript. They participated in the study acquisition, analysis and interpretation of data, and drafting of the manuscript.

### Conflict of Interest

The authors declare that the research was conducted in the absence of any commercial or financial relationships that could be construed as a potential conflict of interest.
